# ViRBase: a resource for virus–host ncRNA-associated interactions

**DOI:** 10.1093/nar/gku903

**Published:** 2014-10-01

**Authors:** Yanhui Li, Changliang Wang, Zhengqiang Miao, Xiaoman Bi, Deng Wu, Nana Jin, Liqiang Wang, Hao Wu, Kun Qian, Chunhua Li, Ting Zhang, Chunrui Zhang, Ying Yi, Hongyan Lai, Yongfei Hu, Lixin Cheng, Kwong-Sak Leung, Xiaobo Li, Fengmin Zhang, Kongning Li, Xia Li, Dong Wang

**Affiliations:** 1 College of Bioinformatics Science and Technology, Harbin Medical University, Harbin, China; 2 Institute of Cardiovascular Sciences and Key Laboratory of Molecular Cardiovascular Sciences, Peking University Health Science Center, Beijing, China; 3 Department of Computer Science and Engineering, The Chinese University of Hong Kong, Hong Kong, China; 4 State Key Laboratory of Emerging Infectious Diseases, The University of Hong Kong, Hong Kong, China; 5 Department of Pathology, Harbin Medical University, Harbin, China; 6 Department of Microbiology, Harbin Medical University, Harbin, China

## Abstract

Increasing evidence reveals that diverse non-coding RNAs (ncRNAs) play critically important roles in viral infection. Viruses can use diverse ncRNAs to manipulate both cellular and viral gene expression to establish a host environment conducive to the completion of the viral life cycle. Many host cellular ncRNAs can also directly or indirectly influence viral replication and even target virus genomes. ViRBase (http://www.rna-society.org/virbase) aims to provide the scientific community with a resource for efficient browsing and visualization of virus-host ncRNA-associated interactions and interaction networks in viral infection. The current version of ViRBase documents more than 12 000 viral and cellular ncRNA-associated virus–virus, virus–host, host–virus and host–host interactions involving more than 460 non-redundant ncRNAs and 4400 protein-coding genes from between more than 60 viruses and 20 hosts. Users can query, browse and manipulate these virus–host ncRNA-associated interactions. ViRBase will be of help in uncovering the generic organizing principles of cellular virus–host ncRNA-associated interaction networks in viral infection.

## INTRODUCTION

Non-coding RNAs (ncRNAs) are important functional RNA molecules (1–3). Recent advances in virology reveal that ncRNAs play critical roles during almost every process of viral infection, such as regulation of virus growth, replication and cell death (2,4,5). Accumulated evidence shows that viral ncRNAs can be used to manipulate both cellular and viral gene expression to establish a host environment conducive to the completion of the viral life cycle (2,6). Similarly, many cellular ncRNAs can also directly or indirectly influence viral replication and even target viral genomes (2,7,8). Interaction of the viral and cellular ncRNAs with their viral and cellular targets forms a complex functional interaction network. Elucidation of such complex interactions is fundamental for understanding viral infection and developing new antiviral therapies.

To facilitate related research in virology, we developed a virus–host ncRNA-associated interaction database (ViRBase; http://www.rna-society.org/virbase), aimed at collecting viral and cellular ncRNA-associated virus–virus, virus–host, host–virus and host–host interactions in viral infection by manually curating the literature. The current version of ViRBase documents over 12 000 viral and cellular ncRNA-associated virus–virus, virus–host, host–virus and host–host interactions involving more than 60 different viruses and 20 hosts. Most medically relevant viruses are contained within the database, including Epstein–Barr virus, human immunodeficiency virus 1 (HIV-1), hepatitis B virus (HBV), hepatitis C virus (HCV), herpes viruses, influenza A virus H1N1 and H3N2, Kaposi's sarcoma-associated herpesvirus, papilloma viruses and simian virus 40. Hence, ViRBase provides a global view of virus–host ncRNA-associated interaction networks in viral infection. Researchers can follow these interactions to explore how the virus–host ncRNA-associated interaction network is organized. The whole data set can be easily queried and downloaded through the webpage, and visualization tools for interactively browsing and analyzing the data set are provided. In addition, ViRBase also allows researchers to submit new virus–host ncRNA-associated interactions.

## DATA SOURCES AND IMPLEMENTATION

In order to collect all available ncRNAs, we have integrated information on three major types of ncRNAs: long non-coding RNA (lncRNA) symbols from lncRNAdb (9) and the functional lncRNA database (www.valadkhanlab.org/database/), microRNA symbols from the mirBase database (10) and small nucleolar RNA (snoRNA) symbols from sno/scaRNAbase (11) and snoRNA-LBME-db (12). We also used the ncRNA category names for other ncRNAs, such as transfer RNA and small nuclear RNA. The list of virus names and abbreviations was collected according to international committee on taxonomy of viruses database (ICTVdb) (13,14). We have written a simple script to screen all abstracts and articles in the PubMed database using the following keyword combinations: (each ncRNA symbol or ncRNA category name) and/or (each virus) and/or (interaction or binding, etc.). The relevant hits were further inspected manually.

The ViRBase database is implemented in the HTML and PHP languages with MySQL server. The interface component consists of webpages designed and implemented in HTML/CSS in a Microsoft Windows environment. It has been tested in the Google Chrome, Firefox and Internet Explorer web browsers.

## CONTENT OF THE DATABASE

**Figure 1.**
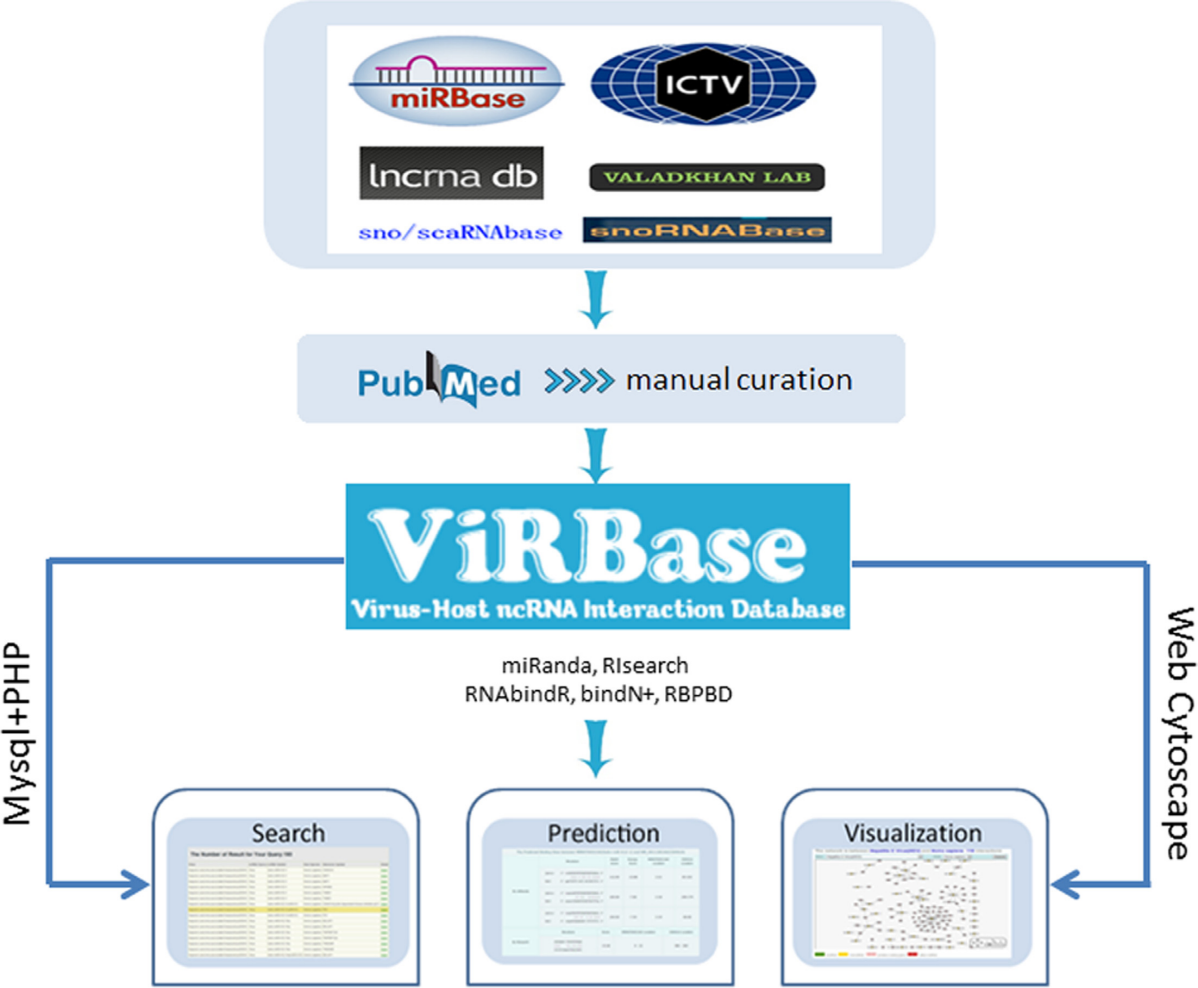
The overview of the ViRBase database.

All interaction information was obtained from articles published in the PubMed database before August 2014. In the current version, ViRBase documents 12 247 ncRNA-associated virus–virus, virus–host, host–virus and host–host interactions in viral infection (including 11 816 experimental interactions and 431 predicted interactions), involving 462 non-redundant ncRNAs and 4463 protein-coding genes from 61 viruses and 24 hosts. Each entry contains detailed information on a virus–host ncRNA-associated interaction, including virus name, host species, ncRNA symbol, target symbol, validation method, Pubmed ID and detailed description (Figure 1).

ViRBase also provides five options on the ‘Help’ page to provide instructions for using the database. These include ‘Tutorial’ (procedure and illustrations of the database), ‘Source’ (sources of ncRNAs and gene information, and tools used in the database), ‘Parameter’ (details the parameters for tools used for binding site prediction), ‘Statistics’ (detailed statistical tables) and ‘Error Report’. In the ‘Download & API’ page, users can download all interaction data in Microsoft Excel and TXT format by selecting ‘ALL Virus–Host Interactions’ or access the application programming interface (API) using scripts. In the ‘Submit’ page, ViRBase invites users to submit novel virus–host ncRNA-associated interactions.

## DATA QUERYING, SEARCHING AND BROWSING

ViRBase provides an interface for convenient retrieval of all interactions. Users can search each virus–host ncRNA-associated interaction through four paths (Supplementary Figure S1), including ‘By keyword’ (search by any key information with support for fuzzy search), ‘By Virus and Host Species’ (select virus in ICTV taxon or host of interest), ‘By RNA/Protein Symbol’ (select RNA/Protein symbols based on the categories with multiple selection supported) and ‘By Validated Methods’ (select or input validated methods of interest; fuzzy search and multiple selections are supported). In the ‘By Validated Methods’ path, we also distinguish between experimentally proven and predicted interactions. Brief details of search results are presented as a table in the ‘Result’ page, while more detailed descriptions such as PubMed ID and description of the reference are displayed in the ‘Detail’ page reached by selecting ‘more’. When selecting the specific RNA/Protein in the ‘Detail’ page (Supplementary Figure S2), the summary page presents more associated information of ncRNA or protein, such as official descriptions, functions (Gene ontology), pathways (KEGG and Biocarta), diseases (OMIM and Genetic Association Database) and interactors (HPRD and BIOGRID).

The ‘Browse page’ enables users to browse the database in four different ways: ‘Browse by Virus’, ‘Browse by Host Species’, ‘Browse by ncRNA Category’ and ‘Browse by ICTV’. All of the virus–host ncRNA-associated interaction information is presented by selecting on each entry.

## VISUALIZATION

**Figure 2.**
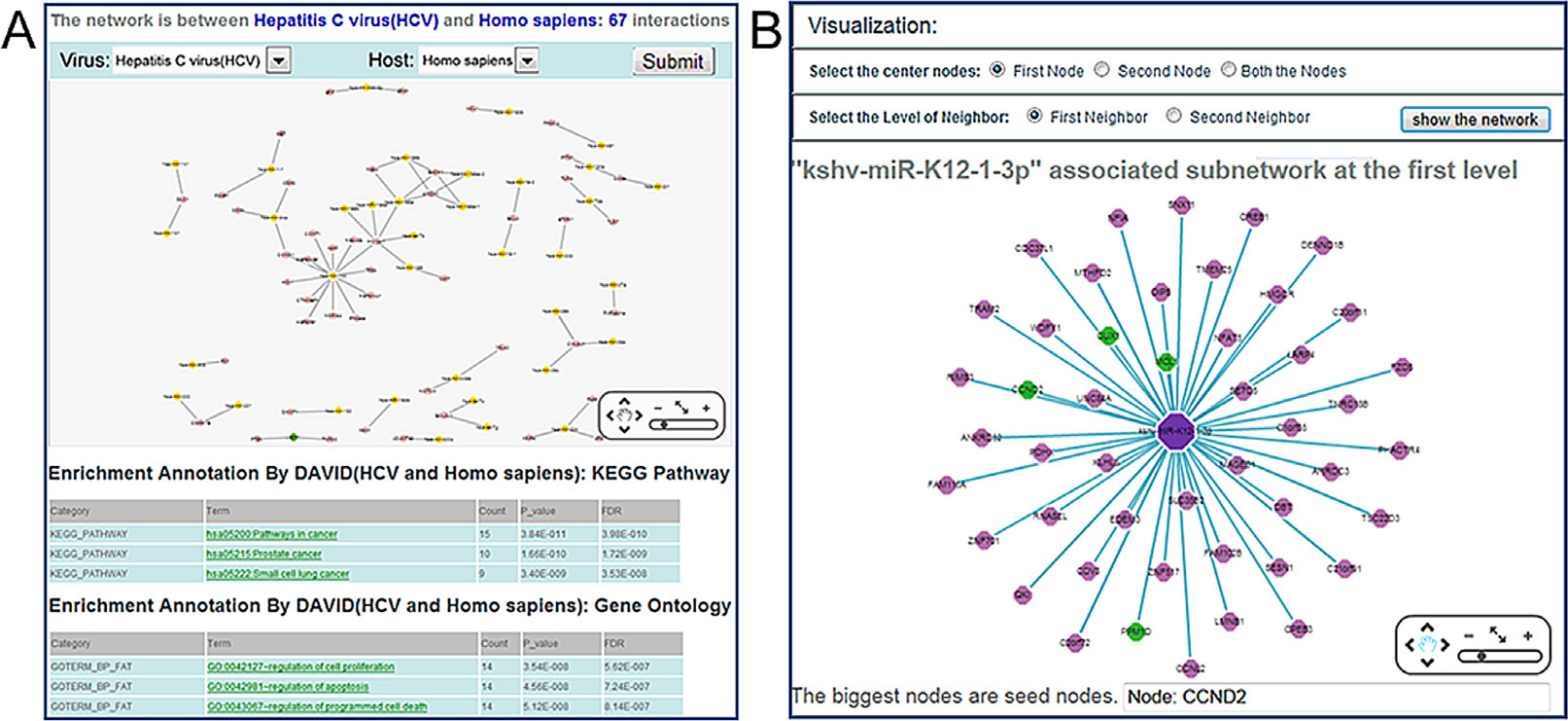
Representative screenshots of the Visualization and Network pages. (**A**) The Visualization page: representing the virus–host ncRNA-associated interaction network for a specific virus and host. At the bottom, the potential Gene Ontology functions and KEGG pathways by performing enrichment analysis are provided. (**B**) The Network page: representing the specific virus–host ncRNA-associated interaction sub-network.

To help users interactively analyze virus–host ncRNA-associated interactions online, ViRBase provides a visualization function by embedding the Cytoscape web tool (http://cytoscapeweb.cytoscape.org/) (15) to highlight/visualize the interaction network or sub-network of a principal interaction. As the compelling visualization architecture is pan-and-zoom, in the ‘Visualization’ page (Figure 2) users can obtain a global view of the virus–host ncRNA-associated interaction network for a specific virus and host. At the bottom of the ‘Visualization’ page, ViRBase also provides the potential Gene Ontology functions and KEGG pathways by performing enrichment analysis of these virus–host ncRNA-associated interaction genes for specific virus and host, including HIV, HBV, H1N1, HCV, etc.

In the visualization option of the ‘Network’ page (Figure 2), virus–host ncRNA-associated interaction sub-networks can also be rapidly and independently represented for specific ncRNA-associated interactions by embedding interactive networks with Cytoscape Web. The ‘First Node’ or ‘Second Node’ option represents the sub-network of interacting RNA/Protein with the first or second interaction RNA/Protein. The ‘Both the Nodes’ option represents the sub-network of interacting RNA/Protein with both interaction nodes. The ‘First Neighbor’ represents the sub-network of direct interaction with the seed node. The ‘Second Neighbor’ represents the sub-network of direct and second-step interactions with the seed node. Interaction sub-networks based on the two nodes of this interaction may help researchers to represent all interacting partners immediately. Thus, multiple RNA/Protein data resources can be combined in a single visualization for each RNA/Protein and its interaction partner. Users can observe specific RNA/Protein pairs within the virus–host ncRNA-associated interaction network and the ‘Selection of the Layout’ option can provide various layout types for this sub-network.

## PREDICTED BINDING SITES

**Figure 3.**
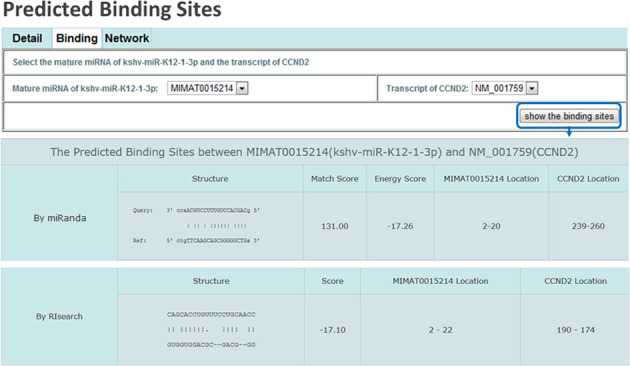
Representative screenshots of the Binding pages. The Binding page represents the predicted binding sites and scores between ncRNAs and their corresponding interactor by various of the predictor tools.

The identification of RNA-associated binding sites can provide valuable insights into the underlying regulatory mechanisms of various ncRNAs, thus the ‘Binding’ page of ViRBase also incorporates several useful tools to analyze the predicted binding site information for these interactions (Figure 3). For ncRNA–RNA interactions, the binding sites and scores are predicted according to miRanda (16) and RIsearch (17). For ncRNA–protein interactions, RNA-binding residues and scores are computed by bindN+ (18) and RNAbindR (19), while the experimentally verified RNA-binding sites documented in RBPBD (20) are also integrated. The parameters used by these tools were documented in the ‘Parameter’ field of the ‘Help’ page.

## DISCUSSION AND FUTURE DIRECTIONS

Several viral databases centered on proteins, such as VirusMINT (21), VirHostNet (22), HIV-1 (23) and the PHISTO (24) database, have been recently constructed and have led to a more comprehensive understanding of viral and host protein function in viral infection. However, viral protein–protein interactions are perhaps only half of the story in viral infection, as increasing numbers of ncRNAs with active involvement in viral infection are reported. Complemented by these useful databases, we developed the ViRBase database by manually collating ncRNA-associated interactions in viral infection. Thus, ViRBase provides an overview of the ncRNA-associated interaction network between viruses and hosts. To our knowledge, this is the first database focusing on ncRNA-associated interactions in viral infection. We hope this resource will bridge the gap in ncRNAs and viral research, and stimulate further interest in exploring the role of diverse ncRNAs in the virus–host network. ViRBase will be of particular interest to the viral science community and will facilitate virologists in unraveling the role of diverse ncRNAs in viral infection. In the future, we also plan to integrate experimentally verified viral protein interactions, providing a more useful resource for a better understanding of the functional organization in viral infection. Finally, we will continue to collate ncRNA-associated interaction reference data and update ViRBase.

## SUPPLEMENTARY DATA

Supplementary Data are available at NAR Online.
